# Proximal myopathy in lacto-vegetarian Asian patients responding to Vitamin D and calcium supplement therapy - two case reports and review of the literature

**DOI:** 10.1186/1752-1947-5-178

**Published:** 2011-05-13

**Authors:** Hood Thabit, Maurice Barry, Seamus Sreenan, Diarmuid Smith

**Affiliations:** 1 Academic Department of Endocrinology, Beaumont Hospital, Dublin, Ireland; 2 Department of Rheumatology, Connolly Memorial Hospital, Dublin, Ireland; 3 Department of Endocrinology and Diabetes Mellitus, Connolly Hospital, Dublin, Ireland

## Abstract

**Introduction:**

Severe proximal myopathy can occasionally be the first presenting complaint of patients with osteomalacia. This may lead to investigations and misdiagnosis of a neuromuscular disease, rather than a metabolic bone disease.

**Case presentations:**

We present here two cases of severe proximal myopathy in patients who were both of South Asian origin and lacto-vegetarians: a 31-year-old Indian man and a 34-year-old Indian woman. In both cases, their clinical symptoms fully resolved following vitamin D and calcium replacement therapy. These patients were at risk of osteomalacia due to their dietary intake and ethnicity. The role of dietary intake and sunlight exposure in the development of osteomalacia in certain ethnic groups living in Western Europe is reviewed here.

**Conclusion:**

These two cases emphasize the importance of recognizing osteomalacia in at-risk individuals, as the condition is reversible and easily treated with vitamin D and calcium supplementation. It may also help avoid prolonged and unnecessary investigations of these patients.

## Introduction

Osteomalacia can present for the first time in some patients as severe muscle weakness and difficulty walking. Proximal myopathy can be present in up to 13% of patients with osteomalacia [1]. This may lead to investigations and misdiagnosis of neuromuscular disease, rather than a metabolic bone disease. It is therefore important in patients known to be at risk of vitamin D deficiency to consider a diagnosis of osteomalacia, as it is easily treatable and reversible. We present here two cases of vitamin D deficiency-induced myopathy.

### Case presentations

#### Case 1

A 31-year-old Indian man, a lacto-vegetarian living in Ireland for five years, presented to our Accident and Emergency department with a two-day history of upper and lower limb tetany. He had a one-year history of increasing bilateral lower limb weakness, which had progressed to the stage where he was not able to stand unaided. He had been referred six months previously to a neurologist, where investigations included a normal magnetic resonance imagining of his brain and spinal cord and negative acetylcholine receptor antibodies. Electromyography studies as well as muscle biopsies of the quadriceps were non-specific (histology showed minimal type 2 fibre atrophy). On examination, he had significant proximal muscle weakness of both lower limbs and a waddling gait. The rest of the clinical examination, including a neurological examination, was normal. Laboratory investigations on admission showed that he was hypocalcemic, with a corrected total serum calcium of 1.43mmol/L (normal range 2.12-2.62mmol/L) and hypophosphatemic, with 0.70mmol/L (normal range 0.8-1.5mmol/L). Serum parathyroid hormone (PTH) (Elecsys 2010 analyser, Roche) was markedly elevated at 595pg/mL (normal range 15-65pg/mL), as was alkaline phosphatase with 254U/L (normal range 38-126U/L). Serum magnesium, renal profile, complete blood count, vitamin B12 and thyroid function tests were all normal. Serum 25-hydroxyvitamin D (Immunodiagnostic Systems, radio-immunoassay) was 5.5nmol/L (seasonal reference range > 50 nmol/L). Plain radiographs of his femur and isotope bone scans were normal. A diagnosis of osteomalacia was made and he was started on ergocalciferol 40,000IU daily for one week, then reduced to twice weekly. He was also started on oral calcium supplements. Following three weeks of therapy his serum calcium level, alkaline phosphatase and PTH levels started to normalize and he was able to walk unaided.

#### Case 2

The second case is of a 34-year-old woman from India living in Ireland for nine years. She was a lacto-vegetarian with no significant past medical history. She complained of aches and pains in her pelvic region for the previous four years and was referred to a rheumatologist complaining of proximal muscle weakness and difficulty walking. Her symptoms transiently improved when she went to back to India for holidays, but reappeared upon returning to Ireland. On physical examination she had significant proximal myopathy and a waddling gait. Serum corrected total serum calcium was low at 2.04 mmol/L with elevated alkaline phosphatase (355U/L) and PTH (104 pg/ml) levels. Her serum 25-hydroxyvitamin D was low (16 nmol/L, seasonal reference range > 50) with a normal fasting serum magnesium and phosphate of 1.18 mmol/L (normal range 0.87-1.45 mmol/L). Complete blood counts, B12, thyroid function tests, erythrocyte sedimentation rate and an auto-antibody screen were all normal. Plain film radiography and isotope bone scans showed no abnormality. As in the first case, a diagnosis of osteomalacia was made based on the clinical and biochemical findings. Medical treatment was initiated, consisting of ergocalciferol 40,000IU once daily for one week followed by twice weekly, together with calcium supplementation. Her serum biochemistry values normalized, together with her clinical symptoms.

## Discussion

Osteomalacia is a disorder of osteoid mineralization characterized biochemically by hypocalcaemia, hypophosphotemia, hypovitaminosis D, raised serum alkaline phosphatase and secondary hyperparathyroidism [2]. Failure to mineralize new bone matrix leads to an increase in both the surface extent and thickness of osteoid seams. However these changes can only be detected on bone biopsies. Osteomalacia is especially prevalent in certain groups of the general population, such as in non-Caucasian immigrants living in Western Europe [3]. In these patients studies have shown that reduced synthesis of 25-hydroxyvitamin D, due to lack of sunlight exposure, and dietary insufficiency of vitamin D appear to be the main causes of osteomalacia [4,5]. The occurrence of osteomalacia can also be related to varying degrees of vegetarianism. Lacto-vegetarians (vegetarian diet which includes dairy products, but excludes eggs) are at greater risk of osteomalacia than ovolacto-vegetarians (vegetarian diet which includes dairy products and eggs) [6]. Both our cases were at increased risk of vitamin D deficiency for two reasons. First, both were South Asians living in a high latitude country where they would have reduced skin production of vitamin D due to higher melanin content, coupled with reduced sunlight exposure. Second, although milk in Ireland is fortified with vitamin D, both patients had limited intake of other dietary sources of vitamin D such as oily fish and eggs. They also consumed unleavened breads, such as chapati, almost daily. Unleavened bread contains phytic acid, which impairs calcium absorption and therefore may account for the severity of presentation of vitamin D deficiency in both our cases [7].

The clinical symptomatology of vitamin D deficiency can vary, but should not be missed by clinicians due to the potential reversibility of the associated symptoms, including myopathy. One of the earliest accounts of osteomalacia associated with profound muscle weakness was by a French surgeon named Jean Louis Petit in 1726. As osteomalacia is the clinical endpoint of vitamin D deficiency, the proximal myopathy observed in these patients presents as a result of this deficiency. The salient features in vitamin D deficiency related myopathy are the proximal distribution, the waddling gait, and pain and discomfort due to muscular effort. Both cases demonstrated these findings clinically. The muscle weakness may develop insidiously over years and patients are frequently referred to different medical specialists in an attempt to make the diagnosis. In our first case the patient had undergone extensive neurological investigations that did not include measurement of his serum calcium or vitamin D levels.

The role of calcium and vitamin D in muscle function may largely explain the profound muscle weaknesses experienced by these patients. It is well recognized that both intra- and extra-cellular calcium are critically important for muscle cell contractility [8]. Experimental studies have also shown skeletal muscle contains vitamin D receptors that specifically bind 1,25(OH)D3 and modulate various transcription factors in muscle cells [9,10]. These factors then mediate muscle cell proliferation and differentiation into mature muscle fibers. Initiation of vitamin D and calcium supplementation once osteomalacia is diagnosed can lead to significant improvements of myopathy and other accompanying symptoms [1]. However, it may take several weeks before the patient's symptoms fully recede. Elevated PTH levels *in vivo *are known to display neurotoxic effects [11]. This may also have contributed to the muscle weakness in our patients. Replacement with ergocalciferol and calcium supplementation resulted in an almost immediate clinical recovery and biochemical normalization of the serum calcium, alkaline phosphatase, PTH and 25-hydroxyvitamin D levels in both of our cases.

## Conclusion

These two case reports highlight the importance of considering vitamin D deficiency in patients presenting with proximal myopathy, especially in those known to belong to high risk groups. A correct diagnosis can help avoid prolonged and needless investigations for these patients, as the condition is reversible and easily treated with vitamin D and calcium supplementation.

## Abbreviations

1,25(OH)D3: 1,25 Dihydroxyvitamin D3; PTH: parathyroid hormone.

## Consent

Written informed consent was obtained from both patients for publication of this case report. A copy of the written consent is available for review by the Editor-in-Chief of this journal.

## Competing interests

The authors declare that they have no competing interests.

## Authors' contributions

HT was responsible for data analysis, the literature search and preparation of the manuscript. MB participated in the data analysis and contributed in the preparation of the manuscript. SS and DS supervised the study and edited the manuscript. All authors have read and approved the final manuscript.
